# Expression and Mutational Analysis of DinB-Like Protein DR0053 in *Deinococcus radiodurans*

**DOI:** 10.1371/journal.pone.0118275

**Published:** 2015-02-23

**Authors:** Deepti Appukuttan, Ho Seong Seo, Sunwook Jeong, Sunghun Im, Minho Joe, Dusup Song, Jungjoon Choi, Sangyong Lim

**Affiliations:** 1 Research Division for Biotechnology, Korea Atomic Energy Research Institute, Jeongeup, Republic of Korea; 2 Department of Agricultural Biotechnology, Center for Agricultural Biomaterials, and Research Institute for Agriculture and Life Sciences, Seoul National University, Seoul, Republic of Korea; Saint Louis University, UNITED STATES

## Abstract

In order to understand the mechanism governing radiation resistance in *Deinococcus radiodurans*, current efforts are aimed at identifying potential candidates from a large repertoire of unique Deinococcal genes and protein families. DR0053 belongs to the DinB/YfiT protein family, which is an over-represented protein family in *D. radiodurans*. We observed that *dr0053* transcript levels were highly induced in response to gamma radiation (γ-radiation) and mitomycin C (MMC) exposure depending on PprI, RecA and the DrtR/S two-component signal transduction system. Protein profiles demonstrated that DR0053 is a highly induced protein in cultures exposed to 10 kGy γ-radiation. We were able to determine the transcriptional start site of *dr0053*, which was induced upon irradiation, and to assign the 133-bp promoter region of *dr0053* as essential for radiation responsiveness through primer extension and promoter deletion analyses. A *dr0053* mutant strain displayed sensitivity to γ-radiation and MMC exposure, but not hydrogen peroxide, suggesting that DR0053 helps cells recover from DNA damage. Bioinformatic analyses revealed that DR0053 is similar to the *Bacillus subtilis* protein YjoA, which is a substrate of bacterial protein-tyrosine kinases. Taken together, the DNA damage-inducible (*din*) gene *dr0053* may be regulated at the transcriptional and post-translational levels.

## Introduction

*Deinococcus radiodurans* (*D. radiodurans*) is a strain of polyextremophilic bacteria that is capable of withstanding up to 15kGy gamma radiation (γ-radiation) [1], several weeks of desiccation [2], 500 J/m^2^ UV-C radiation, [3] and various DNA damaging chemicals such as mitomycin C (MMC) [4], with almost no loss of viability. Furthermore, these bacteria exhibit unmatched resistance to oxidative stress after stimulation with a variety of different stresses [5]. Therefore, the mechanisms that underlie the extreme tolerance to multiple stresses in this organism are primary topics of interest for researchers. Several groups have attempted to ascertain the logistics of this extreme DNA damage resistance and have provided useful insights that aid in elucidating its mechanism [5–8]. However, the exact mechanisms governing the polyextremophilic nature of this bacterial strain still remains unrevealed.

Previous studies have attempted to explain the mechanism of γ-radiation resistance by identifying the roles of radiation-inducible genes. Some novel proteins such as Ddr (DNA damage response) and Ppr (pleiotropic protein promoting DNA repair) are reportedly implicated in the extreme radioresistance of *D. radiodurans* based on the up-regulation of these genes following irradiation and the increased susceptibility of these mutants to γ-radiation [9]. DdrA binds to the 3’ ends of single-stranded DNA to protect them from nuclease degradation [10]. The DdrB protein, which is a prototype of a new bacterial single-stranded DNA-binding protein family [11], stimulates single-stranded DNA annealing [12,13]. These two proteins were recently implicated in an Extended Synthesis-Dependent Strand Annealing (ESDSA)-mediated genome reconstitution process, which is a distinctive DNA repair system in *D. radiodurans* [13,14]. The PprA protein binds to broken double-stranded DNA, protects it from degradation, and stimulates DNA ligase activities *in vitro* [15]. However, recent research has demonstrated that PprA has pleiotropic roles by undergoing dynamic changes in its localization [16]. This protein has been postulated to control DNA segregation during cell division, thus aiding in genome segregation post-DNA double-strand break repair [17,18]. PprI (inducer of PprA) is essential for the extreme radioresistance of *D. radiodurans* [19] and up-regulates more than 200 genes including those involved in DNA repair in response to DNA damage [20]. A transcriptomic study also demonstrated that four genes (*dr0053, dr0841, dr1642*, and *dr1899*) belonging to the DinB/YfiT family were induced in response to γ-radiation. Among them, *dr0053* exhibited the highest induction with approximately 5-fold and 10-fold greater levels in response to 3 kGy and 15 kGy of γ-radiation, respectively [9,21]. *Deinococcus deserti* possesses a homologue of DR0053, Deide_01090, with 47% identity. Deide_01090 was induced more than 50-fold after exposure to γ-radiation [22]. However, the regulation and role of DR0053 under radiation conditions has not been clearly elucidated.

Bioinformatic analysis of the *D. radiodurans* R1 genome has revealed specific expansions of certain protein families compared with other organisms [23]. One of these is the DinB/YfiT protein family. *D. radiodurans* encodes at least 13 DinB/YfiT homologs, which greatly outnumber those found in related Gram-positive bacteria [24]. DinB is a DNA damage-inducible protein, and the *B. subtilis* YfiT protein is induced by general stress [23,24]. Apart from these facts, few studies have explored direct correlations between this protein family and the stress response in *D. radiodurans*. In this study, we investigated the regulatory mechanism underlying *dr0053* expression and examined its role under different abiotic stresses.

## Materials and Methods

### Growth conditions and γ-radiation

*D. radiodurans* R1 (ATCC 13939) was obtained from the American Type Culture Collection (ATCC). *D. radiodurans* was routinely cultivated at 30°C in TGY broth containing 0.5% tryptone, 0.3% yeast extract, and 0.1% glucose. A stationary-phase culture that had been grown overnight (∼14 h) with shaking was used as the seed culture. The seed culture was used to inoculate fresh TGY broth at a 1:100 dilution. For the selection of transformed *D. radiodurans* cells, the medium was supplemented with kanamycin (8 μg/ml) or chloramphenicol (3 μg/ml). Cells grown to log phase (OD_600_ ≈ 1.0) were irradiated at room temperature using a ^60^Co-gamma irradiator (point source, AECL, IR-79; MDS Nordion International Co., Ltd., Ottawa, Canada). The source strength was approximately 215 kCi at a dose rate of 15 kGy/h. Following irradiation, the *Deinococcus* cells were harvested for the subsequent analyses.

### Construction of mutant strains

The *D. radiodurans* mutant strains were constructed using the double cross-over recombination method as previously described [25]. For the construction of the *recA* disruption mutant (*recA*::*cm*), the 1.4-kb fragment containing *recA* was PCR-amplified from *D. radiodurans* genomic DNA using the sequence-specific primer set recA-1F and recA-1R (S1 Table) and cloned into the pGEM-T Easy vector (Promega, Madison, WI). The resulting plasmid was digested with *Nru*I and ligated with the chloramphenicol resistance cassette (916 bp) obtained from the pKatCAT plasmid [26]. The whole ligated product (2,317 bp) was PCR-amplified using the same primer set used for *recA* amplification and transformed into *D. radiodurans* cells as previously described [27]. The chloramphenicol-resistant transformants were grown for several generations in TGY supplemented with chloramphenicol. The *recA* disruption was confirmed by diagnostic PCR using the specific primers recA-2F and recA-2R and nucleotide sequencing (S1 Fig. and S1 Table). Using the primer sets dr0055-1F/dr0055-1R and dr0055-2F/dr0055-2R (S1 Table), approximately 1 kb of the upstream and downstream *dr0055* regions, respectively, were PCR-amplified to construct the *D. radiodurans dr0055* deletion mutant (Δ*dr0055*). The upstream and downstream regions were cloned into the *Xho*I/*EcoR*V and *Xba*I/*Pst*I sites of pKatAPH3 [28], respectively. The resultant recombinant plasmid was transformed into *D. radiodurans* cells, and the transformants were screened on TGY-kanamycin agar plates. Since the sizes of the kanamycin-resistant cassette (*aph*) and *dr0055* gene are very similar, the PCR product obtained from the mutant and wild-type was restriction-digested with *Nde*I to confirm the replacement of the *dr0055* gene with *aph* that has the restriction site for *Nde*I (S1 Fig.). The *D. radiodurans dr0053* deletion mutant (Δ*dr0053*) was constructed as the *dr0055* mutant with some modifications. Briefly, the primer sets dr0053-1F/dr0053-1R and dr0053-2F/dr0053-2R (S1 Table) were used to amplify the upstream (1,852 bp) and downstream (1,856 bp) *dr0053* regions. The dr0053-1R primer is complementary to dr0053-2F; hence, in a second PCR reaction with the dr0053-1F and dr0053-2R primers, the two separate PCR products were combined, generating a fragment lacking the *dr0053* ORF. The final PCR product was cloned into the pGEM T-easy vector (Promega). It was subsequently digested with *Sma*I and then ligated to a 1-kb *Hinc*II fragment harboring the *aph* cassette from pKatAPH3 [28]. This resultant plasmid was transformed into *D. radiodurans* cells, and the transformants were screened on TGY-kanamycin agar plates. Gene replacement was confirmed by diagnostic PCR using the primers dr0053–3F and dr0053–3R (S1 Fig., S1 Table), which bind outside the mutant cassette on the genomic *D. radiodurans* DNA.

### Quantitative real-time PCR (qRT-PCR)

A 5-ml culture grown to log phase (OD_600_ ≈ 1.0) was irradiated or incubated in the presence of MMC (5 μg/ml) or H_2_O_2_ (60 mM) for 1 h. After stress exposure, the cells were collected by centrifugation, re-suspended in 1 ml of RiboEX reagent (GeneAll Biotechnology, Korea) and lysed in a Precellys 24 bead beater (Bertin Technologies, France) using 0.1 mm-diameter Zirconia/Silica beads (Biospec Products, USA). The total RNA was purified using the RNeasy Mini kit (Qiagen, Germany) and RNase-free DNase (Qiagen) according to the manufacturer’s instructions. For real-time PCR analysis, cDNA was synthesized from 1 μg of total RNA using the PrimeScript first-strand cDNA Synthesis Kit (Takara Bio Inc., Japan) as recommended by the manufacturer’s instructions. Real-time qPCR amplification was performed with SYBR Premix Ex Taq (Takara) on an Eco™ Real-Time PCR System (Illumina, USA). The PCR reactions were performed as follows: one cycle of 95°C for 5 m followed by 40 cycles of 95°C for 10 s and 60°C for 30 s. The housekeeping gene *dr1343*, which encodes glyceraldehyde-3-phosphate dehydrogenase, was chosen as the internal control, because its expression level remains unaffected by ionizing radiation [29]. The primers used in for the qRT-PCR assay are summarized in S1 Table. The *D. radiodurans lexA1* (XLK1) and *lexA2* (XL2K1) mutant strains were provided by Dr. K. Satoh of the Japan Atomic Energy Agency, and the *pprI, drtR*, and *drtS* mutant strains were constructed previously and stored in our laboratory [30,31].

### Primer extension assay

The total RNA from the 25 ml of culture was isolated as described in the earlier section (qRT- PCR). The oligonucleotide primer P_dr0053_ (S1 Table) was end-labeled with 80 μCi of [γ-^32^P]-dATP (Amersham Pharmacia Biotech, UK) and 10 units of T4 polynucleotide kinase (Life Technologies, USA) for 30 m at 37°C. The labeling mixture was heated at 70°C for 10 m and purified using MicroSpin G-25 columns (Amersham Pharmacia Biotech). The [γ-^32^P] end-labeled primer (0.5 pmoles) was resuspended in 4 μl of 5× hybridization buffer (1.25 M KCl, 10 mM Tris) and 30 μg of *D. radiodurans* RNA. For hybridization, the mixture was heated to 60°C for 3 m and then cooled to room temperature for 1 h. Subsequently, 5 μl of the reaction solution containing 5 μg of actinomycin D, 700 μM dNTPs, 10 mM MgCl_2_, 5 mM DTT, 20 mM Tris (pH 8.7), 30 units of RNasin (Promega, USA), and 150 units of Superscript Reverse Transcriptase (Life Technologies) was added. The mixture was incubated at 42°C for 70 m and treated with 100 units of RNase T1 (Roche, Switzerland) and 2 μl of 0.5 M EDTA at 37°C for 15 m. The sample was precipitated with ethanol after the addition of 1.4 μl of 5 M NaCl and 2.5 volumes of absolute ethanol and then washed with 75% ethanol. Each sample was resuspended in 6 μl of formamide dye and 4 μl of TE buffer (pH 8.0) and denatured at 90°C for 3 m. To map the first nucleotide of the reaction products, aliquots of each of the reactions were subjected to electrophoresis on 6% polyacrylamide-8 M urea gels alongside sequencing reactions initiated with the same primers that were used for the primer extension reactions. The gels were subsequently dried under a vacuum on filter paper, and the extension products were analyzed on a Fuji Bio-Imaging Analyzer BAS-2500 (Fujifilm, Japan). The primer extension products were run in parallel to the corresponding reactions to map the start site of the *dr0053* transcript.

### Plasmid construction

Six *dr0053* promoter fragments were PCR-amplified using the different set of forward and reverse primers: D53-F1 and D53-R1 for pB1, D53-F2 and D53-R1 for pB2, D53-F2 and D53-R2 for pB3, D53-F3 and D53-R2 for pB4, D53-F3 and D53-R1 for pB5, and D53-F1 and D53-R3 for pB6. These primers are detailed in S1 Table. The pRADZ3 plasmid is an *E. coli-D. radiodurans* shuttle plasmid that harbors a Deinococcal *groESL* promoter driving the expression of the *lacZ* gene [32]. The Deinococcal *groESL* promoter fragment was removed by the restriction digestion of pRADZ3 with *Bgl*II-*Spe*I [25]. Thus, the *Bgl*II-*Spe*I-digested *dr0053* promoter fragments were cloned into *Bgl*II-*Spe*I-digested pRADZ3, thereby replacing the Deinococcal *groESL* promoter with various *dr0053* promoter fragments. Five constructs, pB1 to pB5, were transformed into *D. radiodurans* R1 cells, and the recombinant cells were used for β-galactosidase assay. The pB6 plasmid was transformed into *D. radiodurans dr0053* mutant cells, and the recombinant cells were used for qRT-PCR assay.

### β-galactosidase assay

The β-galactosidase activity of the promoter clones was assessed as previously described [33]. Briefly, recombinant strains grown to log phase (OD_600_ ≈ 1.0) were irradiated and allowed to recover for 1 h at 30°C. A portion of the culture (1 ml) was permeabilized by incubation in cell lysis buffer (10 mM Tris-HCl (pH 8.0), 1 mM EDTA, 100 mM NaCl, 1.5% (w/v) SDS, and 2.5% (v/v) Triton X100) [33] in ice for 10 m. The permeabilized cell suspension was then assayed for β-galactosidase activity using ortho-nitrophenyl-β-galactoside (ONPG) as the substrate as described previously [34].

### Two-dimensional (2-D) gel electrophoresis

*D. radiodurans* cultures grown to log phase (OD_600_ ≈ 1.0) were exposed to 10 kGy of γ-radiation and allowed to recover for 1 h. Subsequently, the cultures were washed with phosphate buffered saline, resuspended in lysis buffer (8 M urea, 2 M thiourea, 2% CHAPS, 1% Pharmalyte, 1% dithiothreitol, and protease inhibitor) and incubated at room temperature for 5 m. The cellular debris was removed by centrifugation (10,000×g/3 m/4°C), and the clear supernatant was precipitated with 100% acetone for 3 h at −20°C. The protein pellet was resuspended in solubilization buffer [9 M urea, 2% β-mercaptoethanol, 2% pharmalyte (pH 4–7), and 8 mM PMSF] and resolved by isoelectric focusing in the first dimension (4–7 IPG strips, Amersham Pharmacia Biotech) using a MultiphorTM II electrophoresis system (GE Healthcare, USA) followed by 12% SDS-PAGE (SE600 Holliston, USA). The protein profile of three biological replicates was analyzed using the PDQuest™ 2-D analysis software (Bio-Rad, USA). The protein spots that differed in intensity by >2-fold in intensity between the control and irradiated samples and passed the t-test control (p<0.05) were identified by peptide mass fingerprinting with the search programs MS-FIT (UCSF Mass Spectrometry Facility, http://prospector.usuf.edu) and Mascot (Matrix Science, London, UK, http://www.matrixscience.com) and the NCBInr and Swiss-Prot databases.

### Survival analysis

Cells grown to log phase (OD_600_ ≈ 1.0) were adjusted to ∼10^7^ CFU/ml in TGY medium and then exposed to different types of DNA-damaging agents. The cells were treated as described below, serially diluted in 0.85% NaCl, spotted on TGY plates, and incubated at 30°C for two days prior to the enumeration of the colonies. For γ-radiation treatment, the cells were irradiated with different doses of γ-radiation at room temperature using a cobalt-60 γ-ray irradiator. The resistance to MMC was determined by exposing *D. radiodurans* cultures to 10 μg/ml MMC in TGY broth. The treated cultures were incubated for 1 or 2 h in the presence of MMC with shaking at 30°C, and the cells were then harvested to determine their viability. For H_2_O_2_ stress treatment, the wild-type and mutant strains were incubated with H_2_O_2_ (0, 40 and 60 mM) with shaking at 30°C for 1 h, and the cell survival fraction was calculated. For UV-stress, the cells were serially diluted in TGY broth and spread on TGY agar plates. After the culture was soaked onto the plates, the plates were exposed to UVC light in a UVC ultraviolet crosslinker (CX-2000, UVP, USA) at 20 J/m^2^/s for different time intervals.

### Purification of DR0053

The genomic DNA from *D. radiodurans* was isolated using the Wizard Genomic DNA purification kit (Promega), according to the manufacturer’s instructions. PCR was performed with the primers (dr0053-4F and dr0053-4R) listed in S1 Table. The PCR product was purified, digested, and ligated into pET28a. The plasmid was then introduced into *E. coli* BL21(DE3) cells by transformation. The DR0053_6XHis_ protein was purified by Ni-NTA agarose (Promega) affinity chromatography, according to the manufacturers’ instructions. In brief, *E. coli* BL21(DE3) cells carrying the pET28a-DR0053 vector were cultured with shaking at 37°C in LB medium (1% tryptone, 0.5% yeast extract, and 0.5% NaCl) containing 50 μg/ml kanamycin to an OD_600_ of 0.8. Protein expression in the cells was then induced by incubating the cells with 0.5 mM isopropyl β-D-1 thiogalactopyranoside (IPTG) for 4 h. The cells were lysed by incubating the cells in 50 mM Tris-HCl (pH 8.0) containing 4 mg/ml lysozyme for 30 m at 37°C. The cellular extracts were then clarified by centrifugation for 30 m at 3800×g, incubated for 2 h with Ni-Agarose beads (GE Healthcare), and applied to a poly-prep column (Bio-Rad). The flow-through was reloaded onto the column and then washed four times with 10 ml of washing buffer (1.5 M NaCl, 25 mM imidazole, and 20 mM Tris, pH 7.5). The bound protein was eluted with 4 ml of binding buffer containing 500 mM imidazole.

### DNase activity assay

The DNase activity of DR0053 was assessed by the DNase agar plate and DNA degradation methods according to previous studies [35]. The purified DR0053_6XHis_ protein (10 μg) or DNase (Qiagen; 1 μg) in PBS (phosphate buffered saline) with 1 mM MgCl_2_ was spotted onto the DNase test agar plates (BD Biosciences, USA). After incubating the plate at 30°C for 5 m, the plate was flooded with 10 ml of 2 N HCl. The excess acid was removed with a vacuum pipette, and the clear zones around DR0053 were compared with DNase as the positive control. The PCR product of the Deinococcal *dr0023* and *dr0024* (3 kb) regions was used to test the direct nuclease activity of the DR0053 protein. The purified PCR product in distilled water with 1 mM MgCl_2_ was incubated with the indicated concentration of DR0053 or DNase for 30 m and the DNA was visualized on a 1% agarose gel.

## Results

### *dr0053* expression is highly induced upon γ-radiation and MMC exposure

**Fig 1:**
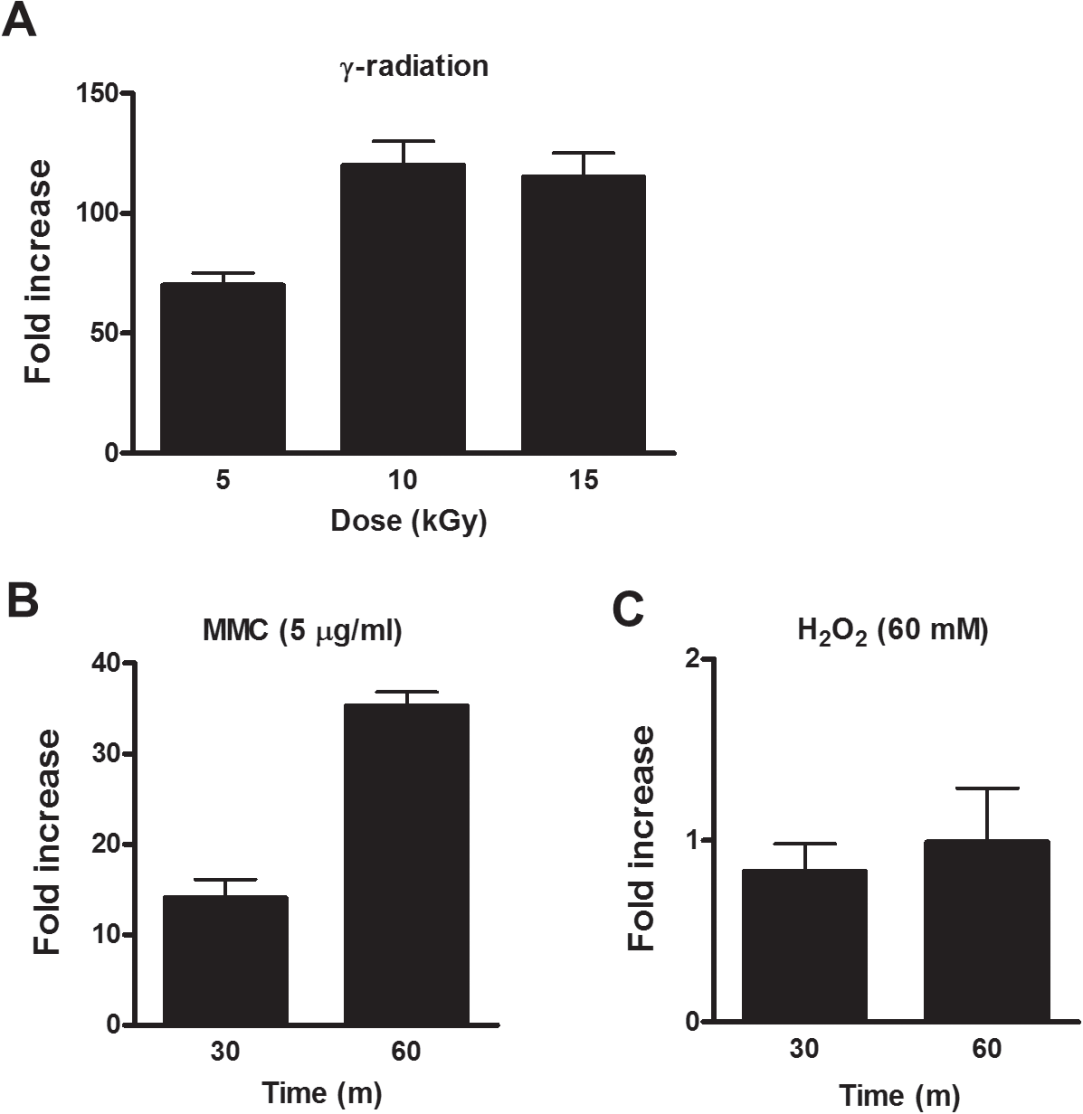
Expression profiles of *dr0053* after γ-radiation, MMC, and H_2_O_2_. *D. radiodurans* R1 cultures were exposed to γ-radiation (A), MMC (B), and H_2_O_2_ (C) at the indicated conditions and were allowed to recover for 1 h. After total RNA isolation, qRT-PCR analysis was performed to determine *dr0053* transcript levels. The fold increase was obtained by dividing *dr0053* expression levels in the treated cells by those from the non-treated cells. Error bars indicate the standard deviations from three independent experiments conducted in duplicate.

The *din* genes in Gram-positive bacteria are induced in response to environmental stressors such as chemical mutagens, radiation, and oxidative stress. Additionally, γ-radiation-induced *dr0053* expression has been previously studied by microarray and quantitative real-time PCR (qRT-PCR) [9,21]. To confirm and examine the pattern of *dr0053* expression under DNA-damage conditions, we performed qRT-PCR using total RNA isolated from wild-type *D. radiodurans* R1 that had been exposed to different doses (0, 5, 10 or 15 kGy) of γ-radiation, MMC (5 μg/ml), or H_2_O_2_ (60 mM). *dr0053* transcript levels remained relatively unchanged immediately after radiation exposure (data not shown). However, there was a marked increase in its expression after 1 h of post-irradiation recovery (PIR) (Fig. 1A). After exposure to 5 kGy, *dr0053* levels were increased by approximately 70-fold compared with the non-irradiated counterpart. These levels were almost doubled after 10 kGy exposure and also remained similar after 15 kGy exposure. *dr0053* induction during PIR is consistent with the previous result that *dr0053* expression peaked 1.5 h after a 15 kGy dose of γ-radiation [21]. *dr0053* expression was also highly upregulated by treatment with another DNA damaging agent (MMC) but not with H_2_O_2_, suggesting that DR0053 is directly or indirectly responsive to DNA damage but not to oxidative stress (Fig. 1B and 1C).

### PprI, RecA, and DrtR are involved in *dr0053* activation

**Fig 2:**
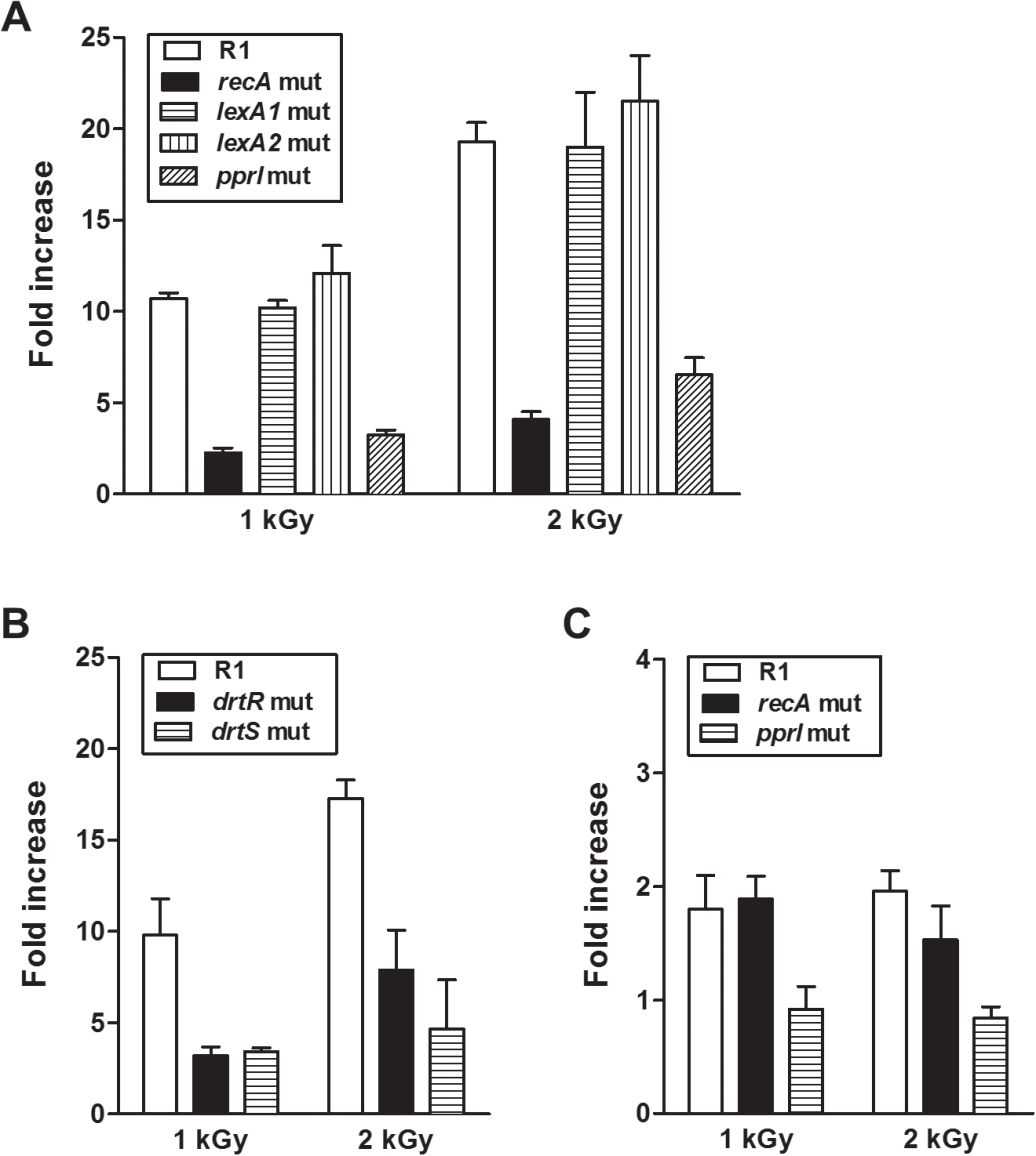
Expression profiles of *dr0053* and *drtR* in mutant strains. Bacterial cultures were exposed to γ-radiation and allowed to recover for 1 h. After total RNA isolation, qRT-PCR analysis was performed to determine *dr0053* (A and B) and *drtR* transcript levels (C). The fold increase in each strain was obtained by dividing the mRNA levels from the irradiated cells by that from the non-irradiated cells. Error bars indicate the standard deviations from three independent experiments performed in duplicate.

The SOS response is a transcriptional circuit that is activated upon DNA damage. The RecA and LexA proteins play key roles in the regulation of the SOS response. RecA activates the SOS response, whereas LexA, a transcriptional repressor, negatively regulates SOS induction [36]. In *B. subtilis, dinB* is a canonical SOS gene; its expression is not activated by DNA-damaging agents in the absence of *recA*, and its promoter has a LexA binding site [37]. Although the error-prone SOS repair system is not observed in *D. radiodurans* due to the absence of *dinP* and *umuC, D. radiodurans* encodes RecA (DR2340) and the two LexA homologues LexA1 (DRA0344) and LexA2 (DRA0074) [5]. We investigated the effects of RecA and LexA on *dr0053* expression using real-time PCR analysis. The marked activation of *dr0053* expression by γ-radiation, which was observed in wild-type strains, was drastically compromised in the *recA* mutant strain (Fig. 2A). The lack of *lexA1* or *lexA2* did not affect *dr0053* expression (Fig. 2A), thus indicating that LexA is not involved in *dr0053* regulation. The PprI protein, a *Deinococcus*-specific regulator, stimulates *recA* transcription and translation following exposure to γ-radiation [19,29]. Similar to the *recA* mutant strain, *dr0053* expression was not fully activated in the *pprI* mutant strain (Fig. 2A). This finding suggests that PprI can affect *dr0053* expression via its regulatory function on RecA.

DrtR (DR2415) and DrtS (DR2416) are a histidine kinase (HK) and a response regulator (RR) of a novel two-component signal transduction system (TCS), respectively, which responds to DNA damage and plays a role in the resistance of *D. radiodurans* to DNA damaging agents [31]. When *dr0053* expression was examined in the *drtR* and *drtS* mutant strains, *dr0053* induction was very poor compared with the wild-type strain (Fig. 2B). Because PprI functions as a general switch to activate DNA repair and various defense pathways [20], we also examined *drtR* expression in the *pprI* mutant strain to investigate the effect of PprI on *drtR*. γ-Radiation-dependent activation of *drtR* expression was observed in the wild-type and *recA* mutant strains, but the activation disappeared in the *pprI* mutant strain (Fig. 2C), indicating that PprI is involved in *drtR* regulation. Additionally, *recA* expression level were not different between the wild-type and *drtR* mutant strains before and after γ-radiation (data not shown). Because RecA and DrtR are necessary for *dr0053* expression and are governed by PprI, PprI appears to be the primary regulator involved in *dr0053* activation by γ-radiation.

### *dr0053* promoter analysis

**Fig 3:**
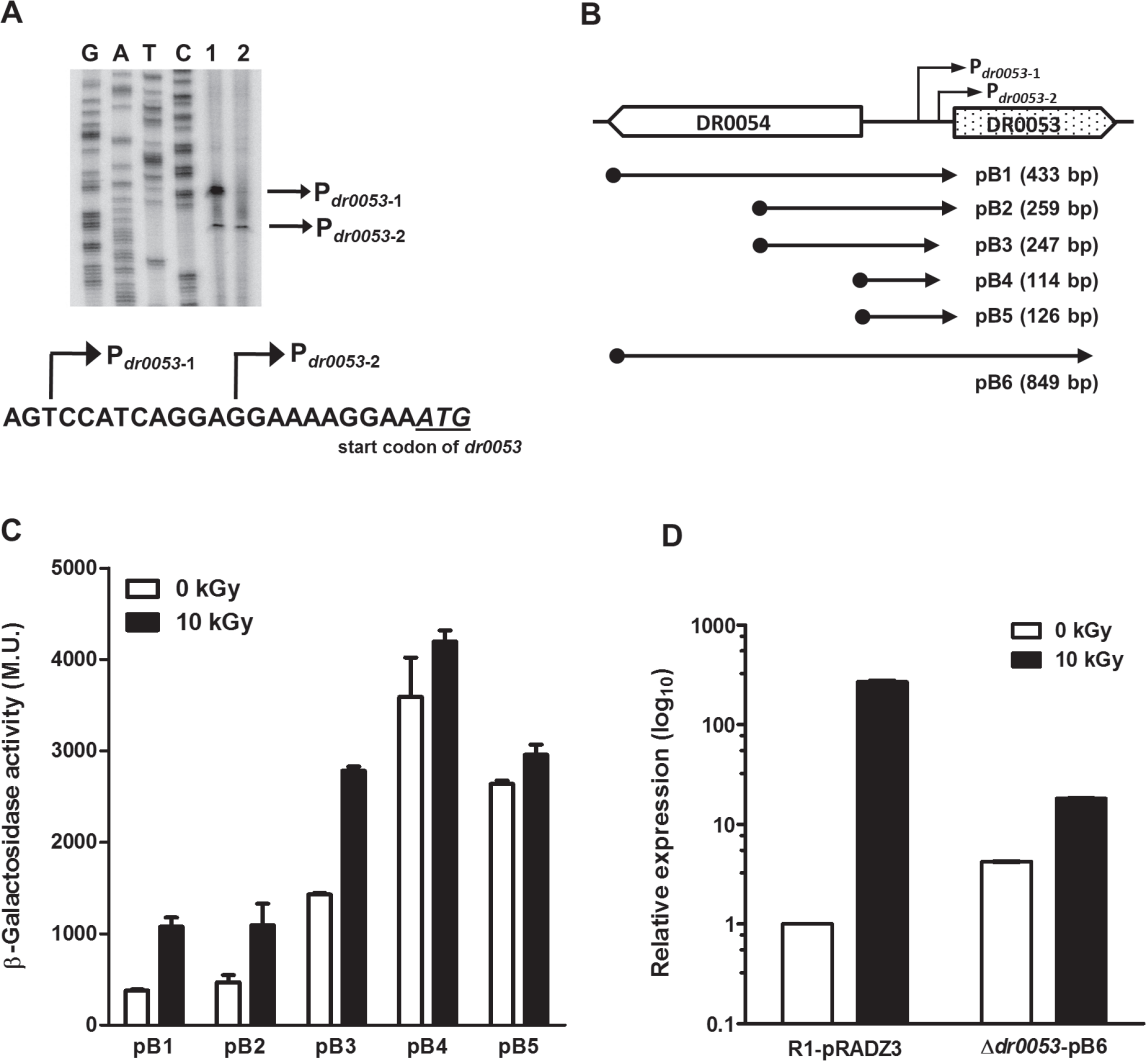
Promoter analysis of *dr0053*. (A) Primer extension assays were performed to map the transcriptional start site of *dr0053*. Total RNAs were extracted from 10 kGy irradiated (lane 1) and non-irradiated (lane 2) *D. radiodurans* cells after recovery for 1 h. RNA aliquots (30 μg) were subjected to primer extension analysis, and a sequence ladder was generated using the same primer. The two putative transcriptional start sites are indicated by arrows. (B) Schematic diagram of the *dr0053* region in the wild-type genome. The coding regions are marked by arrowhead boxes displaying their orientation. The upstream regions of *dr0053* cloned in the pRADZ3 plasmid containing the *lacZ* reporter are aligned below the diagram and are shown next to the plasmid name. The number in the brackets indicates the inserted fragment length. (C) β-galactosidase activities of the R1 cells harboring pB1 to pB5 promoter clones were measured after exposure to γ-radiation followed by 1 h of PIR. The data represent the means and standard deviations from three independent experiments consisting of duplicate samples. (D) The wild-type strains harboring pRADZ3 (R1-pRADZ3) and the *dr0053* mutant strains harboring pB6 (Δ*dr0053*-pB6) were exposed to γ-radiation and were allowed to recover for 1 h. After total RNA isolation, qRT-PCR analysis was performed to determine *dr0053* transcript levels. Relative expression values were determined by defining the mRNA levels from non-treated R1 cultures as 1. Error bars indicate the standard deviations from three independent experiments conducted in duplicate.

*dr0053* transcript levels were highly induced upon γ-radiation (Fig. 1A); therefore, it must possess a strong radiation-inducible promoter. First, we performed a primer extension assay to determine the transcriptional start site of the *dr0053* gene. In total, two primer extension products named P_*dr0053*-1_ and P_*dr0053*-2_ were detected from the total RNA of 10 kGy-irradiated cells. Their corresponding transcription start sites were mapped to positions—20 and—10 nucleotides upstream of the *dr0053* translational start site (Fig. 3A). Next, we performed a promoter deletion analysis to identify the regulatory regions involved in γ-radiation responsiveness. As illustrated in Fig. 3B, various promoter fragments of *dr0053* were cloned into the *lacZ* reporter plasmid pRADZ3 [32]. *D. radiodurans* cells harboring pB1 and pB2 plasmids demonstrated approximately three-fold activation in response to 10 kGy radiation (Fig. 3C). The longer product P_*dr0053*-1_ was induced only after irradiation, whereas the shorter product P_*dr0053*-2_ was expressed constitutively in both irradiated and non-irradiated samples (Fig. 3A). To assess the contribution of P_*dr0053*-2_ to *dr0053* expression, we measured β-galactosidase activity from R1 cells harboring the pB3 plasmid that had only P_*dr0053*-1_. Deletion of the putative –10 region and the transcription start site of P_*dr0053*-2_, which spans the region from –8 to –19 relative to the start codon of *dr0053*, increased *dr0053* expression under both irradiated and non-irradiated conditions, but the γ-radiation-dependent activation of *dr0053* was still observed (Fig. 3C). These results demonstrate that *dr0053* expression is driven by P_*dr0053*-1_ and not by P_*dr0053*-2_. Thus, the shorter product may have been generated by pre-maturation and/or degradation of the longer transcript.

The shortest promoter (pB4) displayed the highest expression of the constructed plasmids, but the β-galactosidase activity remained almost constant under both irradiated and non-irradiated conditions (Fig. 3C). This result indicates that the 133-base-pair (bp) region (from –133 to –266 nucleotides relative to the start codon) between the pB3 and pB4 promoter fragments may provide a binding site for a transcriptional repressor, thereby regulating *dr0053* expression in response to γ-radiation. As the LexA repressor is not involved in *dr0053* expression (Fig. 2A), we examined the proximal region of *dr0053* to search for putative repressor proteins. The *dr0055* ORF, which encodes a repressor protein, is located approximately 441 bp upstream of *dr0053*. To assess whether DR0055 is a repressor of the *dr0053* gene, a *dr0055* deletion mutant was constructed, and *dr0053* expression was examined in this construct using qRT-PCR. There was marginal change in *dr0053* expression in *dr0055* deletion mutant (data not shown), indicating that DR0055 is not a repressor of *dr0053*. Therefore, further research is necessary to determine how *dr0053* is repressed under non-irradiated conditions. When comparing pB2 with pB3, the absence of a 12-bp region increased *dr0053* expression (Fig. 3C). The *dr0053* promoter activity was also reduced in pB5 by extension of the 12-bp region compared to pB4 (Fig. 3C). These results demonstrate that the 12-bp region, which is adjacent to the start codon, plays a role in the repression of *dr0053* regardless of γ-radiation.

The fold increase in activity from the cloned *dr0053* promoters was much lower than its expression from the native genomic site (Figs. 1 and 3). Because the 12-bp region of P_*dr0053*-1_ (positions –8 to –19 relative to the start codon) affected *dr0053* expression (Fig. 3C), sequences around the start of the DR0053 coding region might influence *dr0053* activation in response to γ-radiation. To investigate this possibility, we amplified the DNA fragment, which covers almost the entire DR0054 and DR0053 ORFs, and cloned the 849-bp region into pRADZ3 (Fig. 3B). The pB6 plasmid was introduced into the *dr0053* mutant strains (Δ*dr0053*-pB6), and the fold increase of *dr0053* was measured by qRT-PCR. *dr0053* mRNA levels increased approximately four-fold in Δ*dr0053*-pB6 compared with R1 cells before γ-radiation (Fig. 3D). Following γ-radiation, we observed a 4.5-fold increase in *dr0053* mRNA levels in Δ*dr0053*-pB6, whereas *dr0053* levels increased more than 200-fold in R1 cells (Fig. 3D). This result shows that the low activation of the cloned *dr0053* promoters is not related to a lack of specific sequences surrounding the translational start codon. Because the pRADZ3 plasmid used in this study has copy number similar to that of the chromosome, at 7 to 10 copies per cell [32], this difference in activity may be attributed to the difference in the DNA context surrounding the *dr0053* promoter region in the genome and the plasmid. It is known that DNA supercoiling generated at a local level by transcription can influence nearby events in the same DNA molecule such as promoter activity [38].

### DR0053 is produced at high levels during post-irradiation recovery

**Fig 4:**
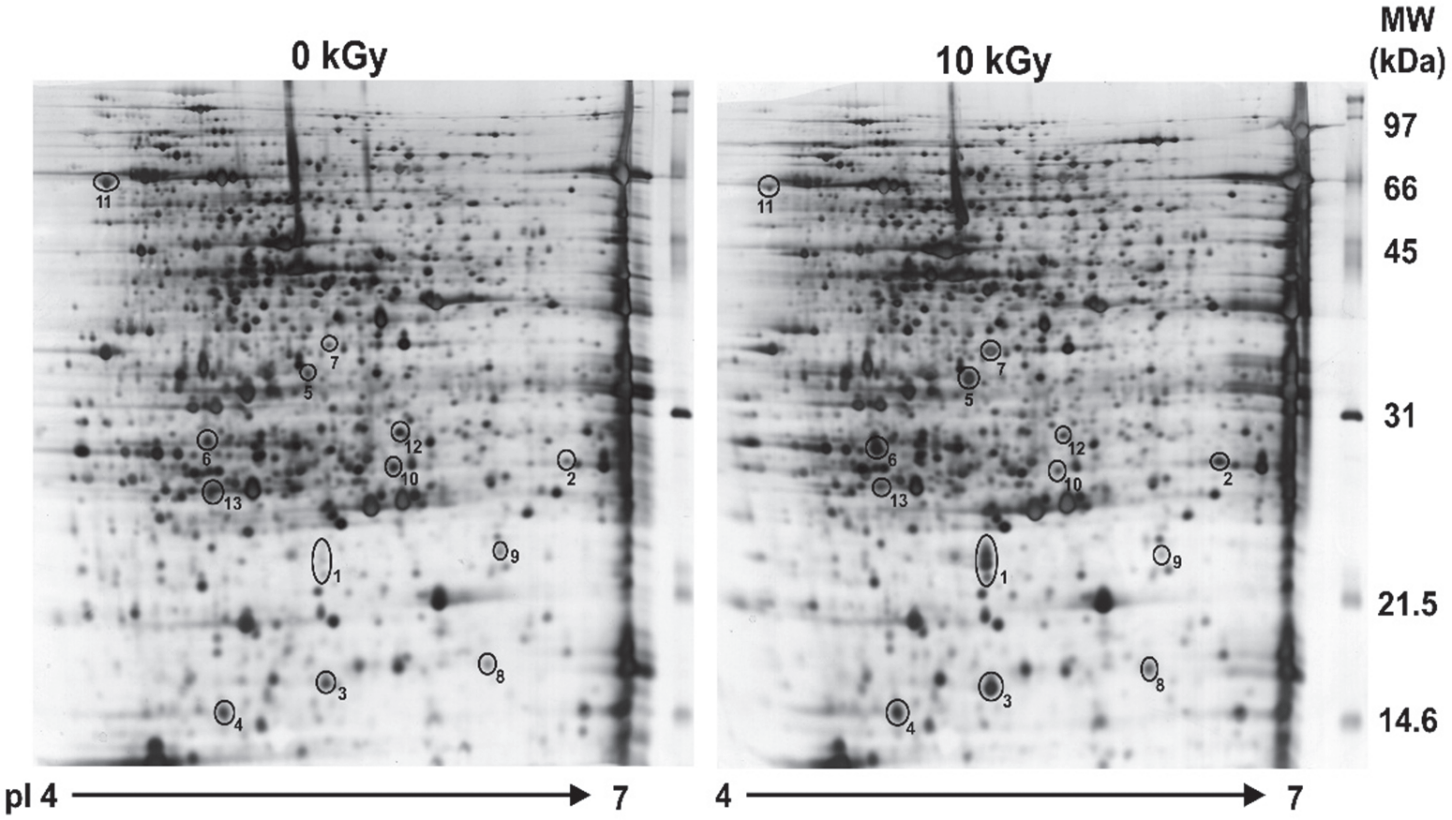
Protein profiles of non-irradiated and irradiated *D. radiodurans*. *D. radiodurans* R1 cultures were exposed to 10 kGy of γ-radiation and allowed to recover for 1 h. Whole-cell proteins were extracted and resolved by isoelectric focusing in the first dimension using 4–7 IPG strips and by 12% SDS-PAGE in the second dimension. The protein spots (marked in circles) that displayed significant differences in expression were selected for further analysis.

When cells encounter any stress, there are often a multitude of changes that occur at the transcriptional level. However, those proteins that are highly critical for stress recovery are preferentially translated into protein. In an effort to determine whether DR0053 is truly induced at the protein level and to identify other DinB/YfiT family of proteins, two-dimensional electrophoresis was performed, and the protein profile of *D. radiodurans* R1 cells exposed to 10 kGy of γ-radiation was compared with that of unirradiated cells after 1 h of PIR. Of the 13 spots that displayed significant changes, eight were up-regulated, and five were down-regulated in response to γ-radiation (Fig. 4 and Table 1). All of these protein spots were identified by MALDI-TOF. DR0053 was among the top induced proteins observed in this 2D protein profile (Fig. 4). In addition, other up-regulated protein spots were identified as DdrA [10], DdrD [9], the single strand binding protein SSB [39], the pleiotropic protein promoting DNA repair PprA [15], and the tellurium resistance protein TerB [40], all of which have been demonstrated to be induced in response to γ-radiation and have been implicated in radiation resistance. However, we were unable to identify any other member of the DinB/YfiT family of proteins in our protein profile.

**Table 1:** List of differentially regulated proteins in R1 cells exposed to 10 kGy of γ-radiation.

**Spot**	**Locus**	**Gene**	**Description**	**M.W. (Da)**	**Score^a^**
**Up-regulated proteins**					
1	DR0053	*dr0053*	DinB/YfiT family protein	18791	76
2	DR0423	*ddrA*	DNA damage response A protein	22988	139
3	DR1857	*osmC*	Alkyl peroxide and oxidative stress response	14521	47
4	DR2220	*terB*	Putative tellurium resistance protein	16608	43
5	DRA0346	*pprA*	DNA damage repair protein	32190	112
6	DR0326	*ddrD*	DNA damage response D protein	21144	150
7	DR0099	*ssb*	Single-stranded DNA binding protein	32702	152
8	DR0556		Conserved hypothetical protein	15798	102
**Down-regulated proteins**					
9	DR0237		Peptidyl-prolyl cis-trans isomerase	21308	75
10	DR1909	*ykgF*	Fe-S protein	22607	280
11	DR1948	*tig*	Trigger factor	51816	247
12	DRA0044		dTDP-4-rhamnose reductase-related protein	26905	102
13	DR0119	efp	Elongation factor P	20462	84

^a^ Individual ions score in MASCOT. The scores>42 indicates identity or extensive homology (p<0.05) in MASCOT search results.

### Gene disruption of *dr0053* increases susceptibility to DNA damage

**Fig 5:**
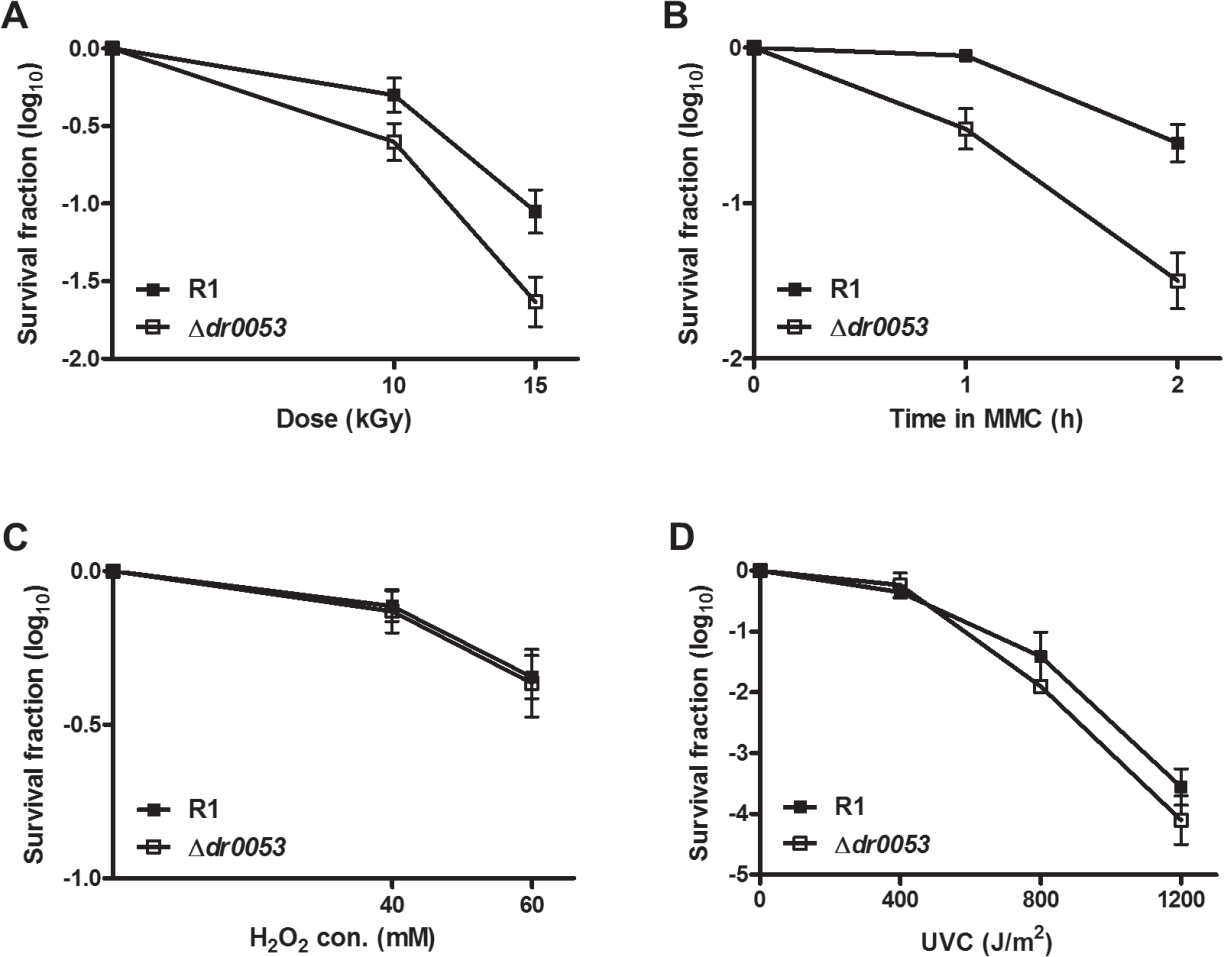
Survival curves for the *dr0053* mutant strain. (A) Irradiated cells (10 and 15 kGy) were plated on TGY plates followed by serial dilution to assess survival. (B) Cells incubated in MMC (10 μg/ml) for 1 and 2 h and (C) incubated in H_2_O_2_ (40 and 60 mM) for 1 h were plated on TGY plates followed by serial dilution. (D) The cells were serially diluted, spotted on TGY agar plates and exposed to UVC radiation. Values are the means and standard deviations from triplicate experiments.

Since *dr0053* was highly induced upon γ-radiation and MMC exposure (Figs. 1 and 4), it may play an important role in survival under DNA-damaging conditions. First, to assess DR0053 function, a *D. radiodurans dr0053* deletion mutant was constructed. The *dr0053* gene was replaced with a kanamycin-resistant cassette (*aph*) under a constitutively expressed Deinococcal catalase (*kat*) promoter. The deletion of *dr0053* did not confer any changes in the growth rate compared with the wild-type strain R1 (data not shown). The *dr0053* mutant strain and *D. radiodurans* R1 were also evaluated for their ability to withstand γ-radiation, MMC, H_2_O_2_, and UVC. Under conditions of 15 kGy γ-radiation, the deletion mutant strain was more sensitive than the wild-type. Similarly, the mutant cells were more sensitive to MMC exposure (at 10 μg/ml) than the wild-type R1 cells. However, no significant differences in survival were observed between the mutant and the wild-type strains in response to H_2_O_2_ and UVC stresses (Fig. 5). The lack of response of the *dr0053* mutant to H_2_O_2_ is consistent with the unchanged expression of *dr0053* after H_2_O_2_ treatment (Fig. 1). This finding suggests that DR0053 may play a role in the survival of *D. radiodurans* under the DNA damaging conditions generated during γ-radiation and MMC treatment.

### DR0053 is a homologue of the *B. subtilis* YjoA protein

**Fig 6:**
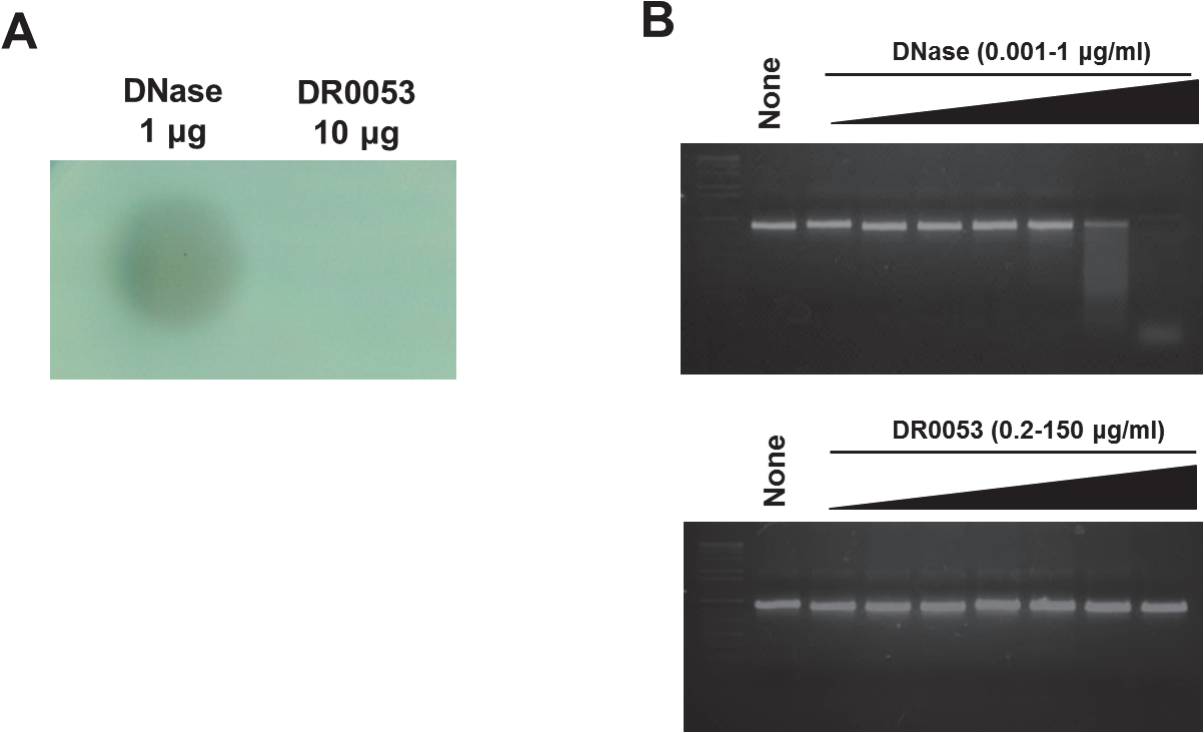
DNase activity assay of DR0053. (A) To assess the DNase activity of DR0053, 10 μl PBS containing DR0053 and 1 mM MgCl_2_ was spotted on DNase test agar. Purchased DNase was used as a positive control. (B) The PCR products (4 μg) amplified from deinococcal genomic DNA were dissolved in 20 μl distilled water containing 1 mM MgCl_2_ and then incubated with the indicated concentration of DR0053 or DNase for 30 m. Samples were subjected to 1% agarose gel electrophoresis and visualized with SYBR-based dyes.

In *D. radiodurans*, it is speculated that DinB-like family proteins are metal-dependent hydrolases because they have three conserved histidine residues, which indicate metal-binding properties [23,24]. Thus, DinB-like proteins are predicted to function as nucleases involved in the cleaning up of DNA damaged products, which are formed immediately after exposure to γ-radiation [23,24]. To investigate whether DR0053 has DNase activity, cell lysates of wild-type and *dr0053* mutant cells were spotted onto DNase test agar according to a previous study [35]. DNase activity was found to be similar in both wild-type and *dr0053* cell lysates, even after exposure to 10 kGy of γ-radiation (data not shown). To examine this DNase activity *in vitro*, we purified the DR0053 protein and compared its activity with that of commercially available DNase I using the same method. A distinct zone of clearance was observed only in the area surrounding commercial DNase I (Fig. 6A). To measure its DNase activity directly, DR0053 was incubated with PCR products amplified from the genomic DNA of R1 cells. However, no detectable DNase activity was seen even when using unusually high concentrations of DR0053 (Fig. 6B). Interestingly, the first and second histidine residues, which are conserved in metal-dependent hydrolases, are substituted with glutamic acid in DR0053 [23].

**Fig 7:**
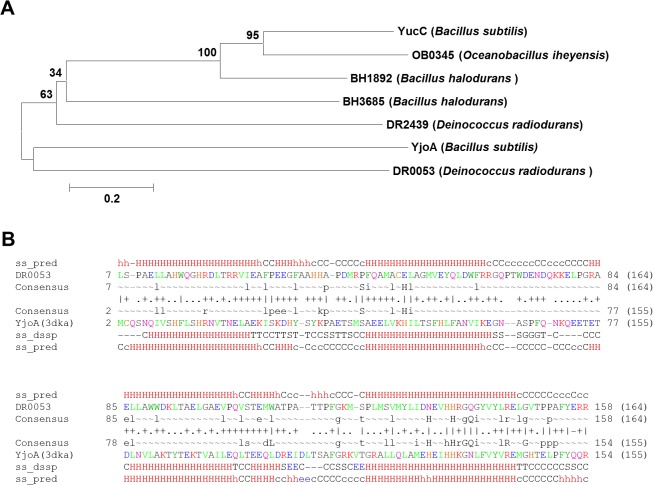
Bioinformatic analyses of DR0053. (A) Phylogenetic tree of protein sequences in the SYSTERS protein family (cluster O154983): DR0053 (Uniprot ID: Q9RY97), BH1892 (Uniprot ID: Q9KBN3), BH3685 (Uniprot ID: Q9K6P3), YucC (Uniprot ID: O05273), YjoA (Uniprot ID: O34334), DR2439 (Uniprot ID: Q9RRP9), and OB0345 (Uniprot ID: Q8ETB6). The sequences were aligned in a ClustalW2 algorithm, and a consensus tree was generated using a 1,000× repeated bootstrapping process with the MEGA 5 program [42]. The numbers on the nodes are the frequency (in percentages) in which a cluster appears in a bootstrap test. (B) Sequence alignment of DR0053 with YjoA (PDB code 3dka) using the HHpred program [44]. Sequences ‘ss_pred’ and ‘ss_dssp’ denote the PSI-PRED secondary structure prediction and the secondary structure assigned by DSSP (Dictionary of Secondary Structure of Proteins) program. Upper and lower case amino acids in the consensus sequences indicate high (≥60%) and moderate (≥40%) conservation, respectively. Red, blue, and black letters represent charged, polar, and hydrophobic residues, respectively. Symbols indicating the quality of the column–column match: ‘|’ very good, ‘+’ good, ‘·’ neutral, and ‘−’ bad.

To gain a better understanding of the structural/functional determinants of the DR0053 protein, a bioinformatic analysis was performed. The pfam domain search demonstrated that DR0053 belongs to the DinB protein family (pfam05163). This family consists of seven SYSTERS (SYSTEmatic Re-Searching) protein families [41]. Among them, DR0053 belongs to the family of cluster O154983 with six other proteins (Fig. 7A). The relationship between DR0053 and the other proteins in this cluster was investigated through neighbor-joining methods of MEGA 5.0 program with 1,000 bootstrap replicates [42]. A phylogenetic tree indicated that DR0053 is located in a distinct phylogenetic branch with YjoA of *B. subtilis* (Fig. 7A). It suggests that DR0053 function might be more similar to YjoA than any other proteins. A structure prediction analysis using PHYRE and HHpred algorithms [43,44] also identified DR0053 as having high structural similarity to the DinB-like protein (YjoA) of *B. subtilis* (HHpred; 100% probability, E-value = 2e-27) (Fig. 7B). These show that DR0053 is a homologue of the *B. subtilis* YjoA protein.

## Discussion

The polyextremophilic *D. radiodurans* encodes 13 DinB/YfiT homologues, which is the highest number identified in any bacteria to date [23,24]. Such particular expansions of certain protein families are favored during evolution to aid in organism survival [23]. Considering the extreme multiple stress tolerance of this bacterium, genes belonging to such protein families may hold essential information to help elucidate its resistance mechanisms. The prototype of Deinococcal DinB family proteins is *B. subtilis* DinB whose expression is controlled by the SOS system [23,37]. In *D. radiodurans*, RecA positively regulated the expression of *dr0053*, one of the DinB family proteins, as it does in *B. subtilis*; however, LexA was not involved in this regulation (Fig. 2A). This result is consistent with the finding that neither of the Deinococcal LexA homologues (LexA1 and LexA2) repressed *recA* expression although they are cleavable by RecA [33,45]. In addition to its participation in the SOS response, *B. subtilis* RecA is also responsible for DNA damage-dependent alterations in gene expression for nearly 600 genes, most of which are not repressed directly by LexA [36]. These observations indicate the presence of another transcriptional repressor, substituting for LexA, which suppresses *dr0053* expression under non-irradiated conditions. Deletion analysis of the *dr0053* promoter demonstrates the possibility that unidentified repressor binding sites are present in the 133-bp region upstream of the transcriptional start site of P_*dr0053*-1_ (Fig. 3). Some regulators are involved in the repression of radiation-inducible genes under non-irradiated conditions. The deletion of *pprM* (DR0907), which encodes a modulator of the PprI-dependent DNA damage response, and *recX* (DR1310) results in constitutive production of PprA and RecA, respectively, regardless of γ-radiation treatment [46,47].

The TCS, which is composed of an HK and an RR, is one of the most ubiquitous means by which bacteria sense, respond, and adapt to environmental changes. The HK perceives the environmental signal and transduces the signal to its cognate RR which, in turn, activates the specific response to adapt the cell to its new surroundings [48]. Until now, three RRs, DrRRA (DR2418), RadR (DRB0091), and DrtR, have been shown to be necessary for radiation resistance in *D. radiodurans* [31,49,50]. Deletion of *drRRA* downregulates the transcriptional levels of numerous genes related to stress response and DNA repair, such as *kat, sod, recA* and *pprA* [49]. Microarray analysis demonstrated that the *drRRA* mutation slightly reduced *dr0053* expression under both normal and irradiation stress conditions [49]. Taking the effect of RecA on *dr0053* expression into consideration (Fig. 2A), DrRRA is likely to have a positive effect on *dr0053* expression via RecA. Here, we also observed that DrtR is necessary for *dr0053* activation by γ-radiation (Fig. 2B), although DrtR was not related to RecA (Fig. 2C). The involvement of two RRs in *dr0053* regulation implies that the function of DR0053 is intimately connected to environmental changes.

The *dr0053* gene was induced in response to γ-radiation in the presence of RecA, DrtR (Fig. 2) and DrRRA [49], and expression of these regulators decreased in the *pprI* mutant strains [19,49]. Therefore, PprI appears to be the primary regulator of *dr0053* and might exert its effect on *dr0053* expression via RecA, DrtR, and/or DrRRA. The *dr0053* gene is found among PprI-dependent genes [20]. However, we observed a three- and six-fold induction in *dr0053* expression in *pprI* mutant strains in response to 1 and 2 kGy of γ-radiation, respectively (Fig. 2A). The double mutant Δ*drRRA*Δ*pprI* was more sensitive to γ-radiation than either the Δ*drRRA* or Δ*pprI* single mutant [51]. In addition, RecA overexpression could partially restore the radioresistance of a *pprI* mutant strain [19]. These findings suggest that PprI and its downstream regulators have non-overlapping routes in addition to a common pathway for the regulation of target genes. Taken together, *dr0053* expression is likely to be finely tuned by a multi-layered regulatory scheme in which PprI, RecA, DrRRA, and/or DrtR act together with unidentified regulatory proteins.

DR0053 has a critical residue found in metal-dependent catalytic enzymes, such as hydrolases and nucleases. Thus, it was previously proposed to encode a nuclease that helps clean up damaged DNA resulting from acute DNA damaging stresses [23,24]. However, when we examined its nuclease activity using the DNase test agar plate and Deinococcal DNA degradation assays according to a previous study [35], no DNase activity was detected under our experimental conditions (Fig. 6). Instead, we found out that DR0053 has a structural conformation that resembles the YjoA protein of *B. subtilis* (Fig. 7). It was recently discovered that YjoA is one of the substrates of the bacterial protein tyrosine-kinase (BY-kinase) PtkA in *B. subtilis* [52]. It should be noted that 9 of 36 (25%) identical amino acid residues between YjoA (153 aa in length) and DR0053 (158 aa in length) are concentrated in the C-terminal tail region (17 aa in length) of each protein. The phosphorylation site of YjoA is Y150 [45]. Thus, the conservation of this segment implies that DR0053 can be a substrate of a homologue of PtkA in *D. radiodurans*, and the Y155 of DR0053 serves as a putative phosphorylation site. Protein phosphorylation is a widespread post-translational modification that plays a key role in the regulation of cellular functions [53]. The eukaryotic-type serine/threonine protein kinase (eSTPK) DR2518, whose synthesis and phosphorylation are induced by γ-radiation, has been characterized for its role in bacterial responses to DNA damage [54]. Recently, PprA was identified as a substrate for this protein kinase [55], suggesting that protein phosphorylation, which can change an enzyme’s activity level, cellular localization, or interaction with partners of the target protein [53], is a defense mechanism in *D. radiodurans*.

DNA repair genes, which are under the control of PprI in response to γ-radiation, exhibit phase-dependent expression during PIR: *recA* is significantly increased at the early and middle phases and return to normal levels in the late phase [20]. In the presence of PprI, interestingly, *dr0053* shows a *recA*-like expression pattern during PIR [20]. In addition, DR0053 was highly produced after γ-radiation (Fig. 4), and the *dr0053* mutant displayed increased sensitivity to γ-radiation and MMC exposure (Fig. 5). Taken together, these observations suggest that DR0053 is necessary to cope with the stress generated from these damaging agents. DR0053 is a probable substrate protein for BY-kinase of *D. radiodurans* (Fig. 7). BY-kinases, which phosphorylate tyrosine residues on their substrate proteins, are involved in several cellular processes, including the heat shock response, DNA replication, and the cell cycle. However, they have been best characterized for their involvement in the production of exopolysaccharide (EPS) [52,56]. A sequence homology search using the PSI-BLAST tool revealed that DRA0033, denoted “ExoP-related protein”, is homologous to PtkA and is located within the gene cluster involved in EPS biosynthesis in *D. radiodurans*. This is consistent with the fact that most experimentally validated BY-kinases are encoded by genes located in large operons involved in EPS biosynthesis and export [53]. Although further research is warranted to identify a link between DR0053 and DRA0033, the location of DRA0033 provides a clue to the role of DR0053.

## Supporting Information

S1 Table Primers used in this study. (DOC)

S1 Fig Verification of gene disruption by PCR. PCR fragments were amplified from genomic DNA of the wild-type R1 (lane 1) and its isogenic mutant strains (lane 2) using the primers listed in S1 Table. PCR products of the *recA* and *dr0053* mutant strains were larger than those of R1 due to the antibiotic marker insertion. The PCR product obtained from the *dr0055* mutant strain was digested with *Nde*I. M denotes the DNA size markers.
(TIF)
