# Supplementary material for: Expression and Mutational Analysis of DinB-Like Protein DR0053 in Deinococcus radiodurans
Source: PLoS One. 2015 Feb 23;10(2):e0118275. doi: 10.1371/journal.pone.0118275 (PMC4338110; doi:10.1371/journal.pone.0118275)
Supplement: S1 Table — (DOC) [file pone.0118275.s001.doc]

| **Primer** | **Sequence (5’ to 3’)** |
| --- | --- |
| **Real-time PCR** | |
| dr1343-RTF | gtgaacgacgagcagtacga |
| dr1343-RTR | gtggtcatgatggccttctc |
| dr0053-RTF | gcatggtggagtaccagctc |
| dr0053-RTR | ctcggcagtcaacttgtcc |
| dr2415-RTF | gacgactacctcaccaagcc |
| dr2415-RTR | ttcttgtcgttcaccgtcag |
| recA-RTF | cgattgatgtggtggtcgt |
| recA-RTR | acctggttgatgaagatggc |
| **Promoter analysis** | |
| D53-F1 | aggggtcgtagatctcggcttgatg |
| D53-F2 | ccatctgcgagatctggtcgtgttc |
| D53-F3 | tcggcttcggagatctgcagcacca |
| D53-R1 | tcgctcatttactagtcctcctgatg |
| D53-R2 | ttaactagtactcagcgtaaaagc |
| D53-R3 | gtaactagtgtggtggacctcgttgtcaa |
| **Primer extension assay** | |
| P_dr0053_ | ctccgggaaagcctcgatgacccggcgggt |
| **Mutant construction** | |
| recA-1F | acgctttaccgctcgatcctgcgta |
| recA-1R | gtcttcacccggacaggggactcg |
| recA-2F | caccgacgacctcgcgtt |
| recA-2R | ccgaagcgtgaacacagtc |
| dr0053-1F | gaattcgaggaagccgtg |
| dr0053-1R | tgagcgacaaactgctgccccggggtaagcgtgccttctttg |
| dr0053-2F | caaagaaggcacgcttaccccggggcagcagtttgtcgctca |
| dr0053-2R | gaccaggtggaaagaatc |
| dr0053-3F | gcgactcggtggtctttcgtg |
| dr0053-3R | accagtttcaggtcggacatg |
| dr0055-1F | tatactcgagacgtcgcgggtgagttcg |
| dr0055-1R | gcgagatatcggcttgatgtacgcaaagc |
| dr0055-2F | ttcatctagagcccttctcctggaggag |
| dr0055-2R | gatactgcagtgacgatgttggcgaggag |
| **DR0053 purification** | |
| dr0053-4F | aggcggccgcaatgagcgacaaactgctgctg |
| dr0053-4R | cactcgagccccagcggcggcaggttgcgg |
