# Supplementary figures and images for: Expression and Mutational Analysis of DinB-Like Protein DR0053 in Deinococcus radiodurans
Source: PLoS One. 2015 Feb 23;10(2):e0118275. doi: 10.1371/journal.pone.0118275 (PMC4338110; doi:10.1371/journal.pone.0118275)

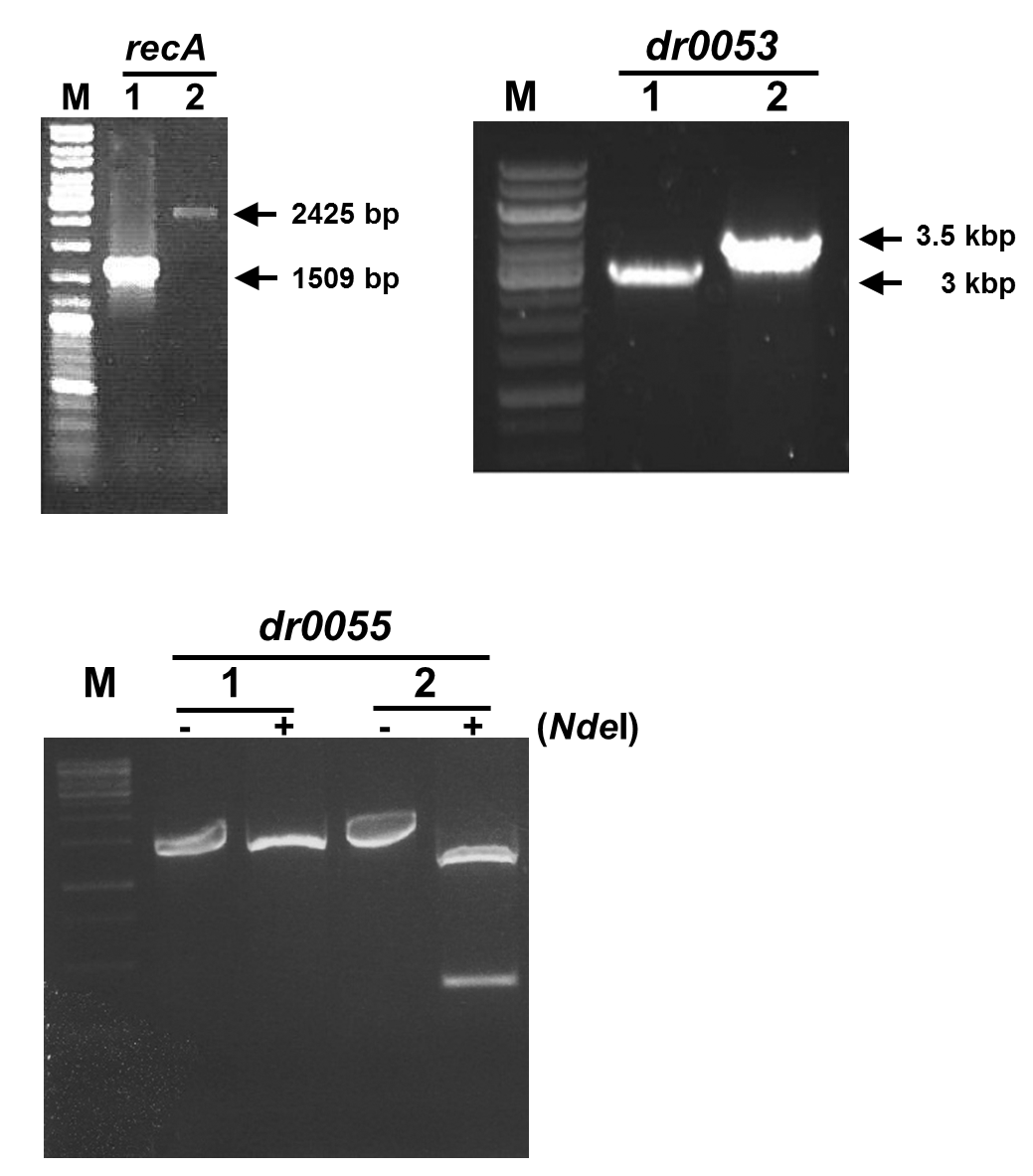

Supplement: S1 Fig — PCR fragments were amplified from genomic DNA of the wild-type R1 (lane 1) and its isogenic mutant strains (lane 2) using the primers listed in S1 Table. PCR products of the recA and dr0053 mutant strains were larger than those of R1 due to the antibiotic marker insertion. The PCR product obtained from the dr0055 mutant strain was digested with NdeI. M denotes the DNA size markers. (TIF) [file pone.0118275.s002.TIF]
